# Burnout among healthcare providers and its association with patient safety management: when exhaustion breaks safety

**DOI:** 10.1186/s40359-026-04890-y

**Published:** 2026-06-05

**Authors:** Hany M. Elshouny, Mahmoud Abdelgawad Elattar, Rasha S. Farag

**Affiliations:** 1 Department of Critical Care Medicine, Al Nahda General Hospital, Makkah Medical Center, Makkah, Saudi Arabia; 2 Department of Intensive Care Medicine, Menoufia Directorate for Health Affairs, Egyptian Ministry of Health and Population, Menoufia, Egypt; 3 Department of Clinical Research, Kafr-Elsheikh Directorate for Health Affairs, Egyptian Ministry of Health and Population, Kafr-El Sheikh, Egypt; 4 Harvard Medical School, Boston, MA USA; 5 Department of Clinical Research, Damietta Directorate for Health Affairs, Egyptian Ministry of Health and Population, Damietta, 34511 Egypt

**Keywords:** Burnout, Healthcare providers, Copenhagen Burnout Inventory, Patient safety, Work schedule

## Abstract

**Introduction:**

Burnout, a pervasive phenomenon among healthcare providers, has been linked to both clinicians’ and patient safety. This study aimed to assess the association between burnout and patient safety.

**Methods:**

This cross-sectional study was conducted at Al Nahda General Hospital in Saudi Arabia from 2023 to 2024. A total of 399 healthcare providers (HCPs) completed an online survey. Burnout was measured using the Copenhagen Burnout Inventory (CBI). The primary outcomes were evaluating the prevalence of burnout among HCPs and measuring its association with Patient Safety Management Activities (PSMA). The secondary outcome was to identify potential risk factors affecting PSMA practice.

**Results:**

Of the study group, 64.4% of HCPs aged 30 to 45 years, 61.2% were physicians, and 50.1% had over 10 years of experience. The prevalence of burnout among participants was 41%. HCPs under 30 years old exhibited a significantly lower prevalence of burnout compared to those aged 30–45 years (Prevalence ratio (PR) = 0.63, 95% CI: 0.46–0.86, *P* = 0.003). Work schedule played a significant role, with night-shift workers showing a higher burnout prevalence compared to those on morning shifts (PR = 1.38, 95% CI: 1.09–1.74, *P* = 0.007). Respondents experiencing burnout showed a significantly higher likelihood of being suboptimal in complying with infection control guidelines (Odds ratio (OR) = 3.78, 95% CI: 1.04–13.69, *P* = 0.04) and were less likely to be familiar with standard precautions for preventing the spread of infections (OR = 0.30, 95% CI: 0.11–0.82, *P* = 0.02) compared to those who were not experiencing burnout. HCPs burnout was significantly associated with higher moderate patient involvement in decision-making (OR = 9.69, 95% CI: 2.53–37.10, *P* = 0.001), speaking up about patient safety concerns within teams and departments (OR = 3.62, 95% CI: 1.18–11.07, *P* = 0.02), and documentation and record-keeping (OR = 5.17, 95% CI: 1.22–21.81, *P* = 0.03). Healthcare setting and depression were significant risk factors associated with reduced PSMA.

**Conclusions:**

HCPs burnout was prevalent among middle-aged staff (aged 30–45 years) and night shift workers. It is potentially associated with patient safety practices, including lapses in infection control, patient involvement, safe communication, and record documentation. Further research is required to include multiple centers and multi-pronged interventions to mitigate burnout and improve patient safety.

**Supplementary Information:**

The online version contains supplementary material available at 10.1186/s40359-026-04890-y.

## Introduction

Burnout is an occupational chronic stress syndrome that is closely associated with healthcare and education professionals who regularly engage with others [1]. It is commonly described through a 3-dimensional key model involving emotional exhaustion, cynicism and detachment from the job, and lack of professional efficacy and work accomplishment [2]. Burnout among healthcare providers (HCPs) has emerged as a growing concern within the healthcare industry [3], with potential negative impacts on patient safety [4]. It is more prevalent among physicians and nurses, particularly those working in emergency departments [5, 6]. Physicians experience substantially higher rates of burnout, with risks reported to be up to 15 times greater than those in other professions. Emergency physicians are especially affected by burnout, reporting moderate to high levels of emotional exhaustion (88.6%) and depersonalization (82.8%) [7]. The global estimate of burnout among the public health workforce is 39% [8]. HCPs burnout reached up to 70% in some healthcare settings in Saudi Arabia [9].

The identified risk factors for HCPs burnout encompass both demographic and workplace-related factors. Demographic predictors feature younger age, female gender, and unmarried status. Key workplace contributors include high workloads and long hours, administrative burden, inadequate staffing, alongside night shifts and low seniority, poor leadership support, inadequate communication, and workplace bullying. Additionally, during COVID-19, frontline exposure, personal protective equipment’s shortage, and emotional stressors like patient deaths amplified vulnerability [7, 10].

Burnout resultant chronic stress can lead to adverse consequences for both the providers themselves and the patients they serve, ultimately compromising the efficiency and quality of medical services provided [11]. It is associated with worsening patient safety, with a probability of superiority reaching up to 66.4% [12]. Recent Saudi study supports an organizational pathway linking workplace conditions, burnout, and patient safety. It reported that hospital work conditions, including leadership support, physician engagement, and workload, were associated with perceived patient safety and adverse event reporting [13]. Extensive research has been conducted to investigate burnout within healthcare occupational groups. However, the significance of HCPs burnout on patient safety and associated risk factors varies widely between healthcare settings [14]. Despite established prevalence and general safety associations, studies rarely quantify burnout simultaneously with setting-specific patient safety metrics while identifying modifiable risk factors and interventions. This study aimed to evaluate the prevalence of burnout among HCPs, investigate its association with aspects of patient safety within healthcare settings, and identify the potential risk factors and probable recommended interventions to mitigate burnout and improve patient safety.

## Methods

### Study design

We conducted a cross-sectional study at Al Nahda General Hospital in Saudi Arabia over 6 months, from October 2023 to April 2024, using an anonymous self-administered electronic questionnaire. The survey was in English to better align with the medical field and the professional background of healthcare providers, ensuring clarity and consistency in responses. The survey covered three sections. First, demographic characteristics of HCPs including age, gender, the highest level of education, profession/specialty, years of experience, healthcare setting, work schedule, number of night shifts per week, marital status, number of children, chronic diseases, and annual income. Second, the assessment of HCPs burnout. Third, Patient Safety Management Activities (PSMA).

We adhered to Strengthening the Reporting of Observational Studies in Epidemiology (STROBE) guidelines.

### Participants and settings

The study population consisted of a diverse group of HCPs; physicians, pharmacists, nurses, physical therapists, dentists, chemists, and medical support staff working in various healthcare settings; including hospital wards, outpatient clinics, pharmacies, long-term care facilities, laboratory and technical offices of Al Nahda General Hospital, a tertiary hospital with around 200 beds.

### Ethical considerations

The study was conducted in accordance with the Declaration of Helsinki. Ethical considerations included obtaining institutional review board approval from Al Nahda General Hospital Research and Quality Committee in October 2023 and online informed consent from participants. The participants were informed about the study’s purpose and procedures, ensuring confidentiality and anonymity of data, and following ethical guidelines for research involving human subjects. Participation was voluntary, and respondents could withdraw at any time.

### Exposure and outcomes

We assessed burnout using the Copenhagen Burnout Inventory (CBI) [15, 16], and assessed mental health using the Depression, Anxiety, and Stress Scale (DASS) [17]. The CBI is one of the most widely used and validated tools for burnout assessment. It consists of three subscales with 19 questionnaire items assessing different aspects of burnout: personal burnout, occupational/ work-related burnout, and client-related burnout. Personal burnout (6 items) evaluates prolonged physical and psychological exhaustion that accumulates throughout the day, regardless of occupation. Work-related burnout (7 items) measures exhaustion specifically caused by professional responsibilities. Client-related burnout (6 items) assesses exhaustion linked to interactions with clients, patients, or customers. Each subscale is scored on a scale from 0 to 100, with higher scores indicating greater burnout levels. Each item is rated on a 5-point Likert scale, with varying response options depending on the subscale. For personal burnout, questions assess general exhaustion, such as feeling physically and emotionally drained, worn out, or weak. Responses range from Always (100 points) to Never (0 points); the total score is the average of all items. If fewer than three items are answered, the respondent is classified as a non-responder. Work-related burnout items evaluate emotional exhaustion, frustration, morning fatigue, and energy levels outside work. It follows a similar scoring system but includes both degree-based and frequency-based response options. The first three questions assess burnout on a scale from “To a very high degree (100 points)” to “To a very low degree (0 points),” while the last four questions follow the frequency-based system. If fewer than four questions are answered, the respondent is considered a non-responder. Client-related burnout questions focus on emotional strain, frustration, and exhaustion due to client interactions. It follows a mixed scoring system similar to the work-related burnout scale, with degree-based responses for the first four items and frequency-based responses for the last two. If fewer than three items are answered, the respondent is classified as a non-responder. The total burnout score is the average of all three subscales. Participants were categorized according to burnout status, burnout vs. no burnout, with scores ≥ 50 considered indicative of burnout. Burnout severity is classified as moderate (50–74), high (75–99), and severe (100) [18].

The DASS is a self-report questionnaire designed to assess the mental health conditions and the severity of symptoms related to depression, anxiety, and stress. It involves 21 questionnaire items, each of which is answered on a 4-point Likert scale, ranging from 0 (did not apply to me at all) to 3 (applied to me very much or most of the time).

The primary outcomes: to evaluate the prevalence of burnout among HCPs and its association with Patient Safety Management Activities (PSMA). PSMA referred to healthcare safety-related practices and perceptions, and were assessed using the standards specified by the Joint Commission on Accreditation of Healthcare Organizations (JCAHO) [19]. We created a set of assessment criteria based on the JCAHO standards. This set included ten domains of National Patient Safety Goals (NPSGs): correct patient identification, medication safety, infection prevention and control, patient communication and education; adherence to evidence-based clinical guidelines, patient safety culture, performance improvement and quality initiatives, patient rights and privacy, emergency preparedness, and documentation and record-keeping. Each standard will be assessed as an ordinal variable of 3 categories; “Yes”, “No”, or “Sometimes/Maybe/ Neutral”. Neutral responses represent a moderate, inconsistent or suboptimal rather than optimal performance, and not as equivalent to poor performance. This authors-developed self-reported PSMA assessment tool was developed specifically for this study and systematically grounded in the JCAHO NPSGs as an internationally recognized, evidence-based standards developed through rigorous expert consensus, providing a structured and clinically credible framework for assessing PSMA across the ten domains examined and serving as an operational definition of its practice, and anchoring context-specific assessment items in an established regulatory framework as a recognized methodology in health services research when no single validated instrument adequately captures the setting-specific safety practices under investigation as noted by DeVellis (2016) in Scale Development [20].

The secondary outcomes were to identify the potential risk factors affecting practicing PSMA and probably recommend interventions to mitigate burnout and improve patient safety.

### Sample size

Based on a population size of 1500, a confidence level of 95%, a margin of error of 5%, and a burnout population proportion of 50% [21], the required sample size was determined to be 306 participants. To account for an anticipated 25% non-response rate, the target sample size was adjusted to 408 participants. The sample size was calculated using an online sample size calculator [22]. A simple random sampling technique was used to gather responses from participants.

### Data collection

HCPs were invited to participate in the study via institutional emails, which included a brief explanation of the study objectives, the voluntary nature of participation, and a link to the online Google Form survey (Supplemental content). As for the random sampling method, we ensured a fair and representative selection of participants. Email invitations were sent to a randomly selected sample of 410 healthcare providers. The survey was conducted using an anonymous, self-administered electronic questionnaire, ensuring that no identifying personal information was collected. Data collection was managed by the research team without any involvement from hospital administration or supervisors to prevent any potential bias or pressure. Up to four reminder emails were sent at one-week intervals to encourage participation while emphasizing that responses were voluntary.

### Statistical analysis

Quantitative data were analyzed using descriptive HCPs burnout and patient safety using R, version 4.1.3 (R Foundation for Statistical Computing) [23]. Descriptive statistics were performed to compare the baseline demographics and clinical characteristics of healthcare workers with burnout versus non-burnout. Categorical variables were reported as frequency (percentage) and were compared using the Pearson’s chi-squared (X^2^) test. Continuous variables were presented as mean (standard deviation; SD) if normally distributed and were compared using Student’s t-test. Two-sided p-value < 0.05 were considered statistically significant.

We used Poisson regression models to estimate prevalence ratio (PR) of burnout and 95% confidence intervals (CI) comparing HCPs of burnout versus not. Modified Poisson regression analysis aimed at assessing the adjusted effect of each potential factor contributing to burnout among HCPs while controlling for the other factors.

Statistical models were built based on the potential confounders for the association between burnout and patient safety. They included age, gender, level of education, specialty, experience, healthcare setting, work schedule, number of night shifts per week, chronic diseases, number of children, annual income, and DASS.

Univariable ordinal logistic regression was used to estimate the crude odds ratio (OR) for the association between healthcare worker burnout and patient safety and quality of care with 95% confidence intervals (95%CI). Multivariable ordinal logistic regression was used to adjust for demographic and clinical characteristics and other potential confounders. Poisson and logistic regression models’ assumptions were assessed prior to analysis, and model stability was evaluated to ensure the robustness and credibility of the results.

Given that analyses were conducted across ten PSMA domains, the potential for inflated Type I error due to multiple comparisons was considered. A two-sided p-value threshold of < 0.05 was applied consistently across all models, and only associations that remained significant following multivariable adjustment were emphasized in the interpretation of findings. Variance Inflation Factor (VIF) values were examined prior to multivariable modeling to assess multicollinearity among covariates; no evidence of problematic collinearity was identified (VIF < 5 for all covariates), including between burnout (CBI) and mental health (DASS) variables.

Missing data were minimal and handled using a complete-case per-analysis approach, whereby each analysis included all participants with non-missing data for the variables involved. No imputation methods were applied, as the proportion of missing values for individual variables was low and unlikely to meaningfully influence the estimates. We assessed the pattern of missingness and found no evidence suggesting systematic or differential missingness across key study variables.

## Results

This study included 399 HCPs who responded to the invitations out of 410 invited participants (response rate: 97.3%). The majority of participants were male (62.4%), physicians (61.2%), with the largest age group being individuals aged 30 to 45 years (64.4%). Approximately 33.6% of the participating HCPs held master’s degrees. More than half had over 10 years of experience (50.1%). The prevalence of chronic diseases among respondents was notable (19.3%), with hypertension (HTN) being the most common (6.0%). The mental health status, as assessed by the DASS Scale, revealed that the included HCPs experienced depression (77.4%), anxiety (71.2%), and stress (94.0%). There was a significant difference between those who were experiencing burnout versus those who were not in terms of age, night work schedule, marital status, personal burnout, work-related burnout, client-related burnout, depression, anxiety, and stress. Table 1 presents an overview of the surveyed healthcare providers, providing insights into various demographic, educational, and occupational characteristics.

**Table 1:** Baseline Demographic, educational, occupational, and clinical characteristics of the surveyed healthcare professionals based on burnout status

Characteristics	Total (*N* = 399)	Burnout (*N* = 164)	No burnout (*N* = 235)	*P* value†
Age categories				
< 30 years	113 (28.3)	34 (20.7)	79 (33.6)	**0.007**
30 to 45 years	257 (64.4)	123 (75.0)	134 (57.0)	**< 0.001**
> 45 years	29 (7.3)	7 (4.3)	22 (9.4)	0.08
Gender (Male)	249 (62.4)	110 (67.1)	139 (59.1)	0.13
Highest level of education				
Diploma	11 (2.8)	1 (0.6)	10 (4.3)	**0.03**
Student	16 (4.0)	8 (4.9)	8 (3.4)	0.63
Bachelor	178 (44.6)	67 (40.9)	111 (47.2)	0.25
PGDip	7 (1.8)	2 (1.2)	5 (2.1)	0.71
Fellowship	53 (13.3)	26 (15.9)	27 (11.5)	0.27
Master’s degree	134 (33.6)	60 (36.6)	74 (31.5)	0.34
Specialty				
Physician	244 (61.2)	108 (65.9)	136 (57.9)	0.13
Nurse	129 (32.3)	48 (29.3)	81 (34.5)	0.33
Pharmacist	22 (5.5)	8 (4.9)	14 (6.0)	0.81
Others ^a^	4 (1.1)	0 (0.0)	4 (1.7)	0.15
Experience				
> 10 years	200 (50.1)	90 (54.9)	110 (46.8)	0.14
5–10 years	99 (24.8)	39 (23.8)	60 (25.5)	0.78
< 5 years	100 (25.1)	35 (21.3)	65 (27.7)	0.19
Healthcare setting				
Hospital ward/s	349 (87.5)	147 (89.6)	202 (86.0)	0.35
Outpatient clinic/s	22 (5.5)	7 (4.3)	15 (6.4)	0.49
Pharmacy	7 (1.8)	1 (0.6)	6 (2.5)	0.65
Long-term care facility	4 (1.0)	1 (0.6)	3 (1.3)	0.25
Others ^b^	6 (1.6)	1 (0.6)	5 (2.1)	0.41
Work schedule				
Morning	332 (83.2)	141 (86.0)	191 (81.3)	0.45
Afternoon	182 (45.6)	83 (50.6)	99 (42.1)	0.25
Night	168 (42.1)	82 (50.0)	86 (36.6)	**0.03**
Number of night shifts per week				
> two	148 (37.1)	61 (37.2)	87 (37.0)	1.00
Two	124 (31.1)	54 (32.9)	70 (29.8)	0.58
One	103 (25.8)	35 (21.3)	68 (28.9)	0.11
None	22 (5.5)	12 (7.3)	10 (4.3)	0.27
Marital status				
Married	268 (67.2)	125 (76.2)	143 (60.9)	**0.002**
Divorced	10 (2.5)	4 (2.4)	6 (2.6)	1.00
Single	113 (28.4)	30 (18.3)	83 (35.3)	**< 0.001**
Number of children				
> two	139 (34.8)	64 (39.0)	75 (31.9)	0.17
One - two	127 (31.8)	59 (36.0)	68 (28.9)	0.17
None	133 (33.3)	41 (25.0)	92 (39.1)	0.004*
Annual income				
Less than 50k SAR	204 (51.1)	76 (46.3)	128 (54.5)	0.14
Between 50k to 100k SAR	98 (24.6)	42 (25.6)	56 (23.8)	0.77
More than 100k SAR	81 (20.3)	40 (24.4)	41 (17.4)	0.12
Chronic diseases	77 (19.3)	36 (22.0)	41 (17.4)	0.32
Pre-diabetes mellitus	6 (1.5)	1 (0.6)	5 (2.1)	0.41
Diabetes mellitus	20 (5.0)	10 (6.1)	10 (4.3)	0.49
Hypertension	24 (6.0)	12 (7.3)	12 (5.1)	0.40
Dyslipidemia	3 (0.8)	2 (1.2)	1 (0.4)	0.57
Asthma	7 (1.8)	3 (1.8)	4 (1.7)	1.00
Hashimoto thyroiditis	6 (1.5)	3 (1.8)	3 (1.3)	0.69
Chronic heart diseases	3 (0.8)	2 (1.2)	1 (0.4)	0.57
GERD	4 (1.0)	2 (1.2)	2 (0.9)	1.00
Hypothyroidism	1 (0.3)	0 (0.0)	1 (0.4)	1.00
Obstructive sleep apnea	2 (0.5)	2 (1.2)	0 (0.0)	0.17
Obesity	1 (0.3)	1 (0.6)	0 (0.0)	0.41
Psoriasis	2 (0.5)	1 (0.6)	1 (0.4)	1.00
Multiple sclerosis	2 (0.5)	1 (0.6)	1 (0.4)	1.00
Varicose veins	3 (0.8)	2 (1.2)	1 (0.4)	0.57
Copenhagen Burnout Inventory domains ^c^				
Personal burnout, mean ± SD	19.1 ± 14.5	31.8 ± 13.2	10.2 ± 6.6	**< 0.001**
Moderate	237 (59.4)	31 (18.9)	206 (87.7)	**< 0.001**
High	49 (12.3)	20 (12.2)	29 (12.3)	1.00
Severe	113 (28.3)	113 (68.9)	0 (0.0)	**< 0.001**
Work-related burnout, mean ± SD	8.4 ± 7.6	14.5 ± 8.2	4.1 ± 2.7	**< 0.001**
Moderate	161 (40.4)	19 (11.6)	142 (60.4)	**< 0.001**
High	116 (29.1)	23 (14.0)	93 (39.6)	**< 0.001**
Severe	122 (30.6)	122 (74.4)	0 (0.0)	**< 0.001**
Client-related burnout, mean ± SD	22.8 ± 14.5	26.7 ± 12.5	20.1 ± 15.3	**< 0.001**
Moderate	82 (20.6)	36 (22.0)	46 (19.6)	0.65
High	35 (8.8)	20 (12.2)	15 (6.4)	0.07
Severe	282 (70.7)	108 (65.9)	174 (74.0)	0.09
DASS ^d^				
Depression	6.5 ± 4.4	8.8 ± 4.3	4.8 ± 3.6	**< 0.001**
Normal	309 (77.4)	97 (59.1)	212 (90.2)	**< 0.001**
Mild	63 (15.8)	46 (28.0)	17 (7.2)	**< 0.001**
Moderate	24 (6.0)	18 (11.0)	6 (2.6)	**0.001**
Severe	3 (0.8)	3 (1.8)	0 (0.0)	0.07
Anxiety	5.8 ± 4.6	8.0 ± 4.9	4.3 ± 3.7	**< 0.001**
Normal	284 (71.2)	90 (54.9)	194 (82.6)	**< 0.001**
Mild	46 (11.5)	24 (14.6)	22 (9.4)	0.14
Moderate	51 (12.8)	34 (20.7)	17 (7.2)	**< 0.001**
Severe	12 (3.0)	10 (6.1)	2 (0.9)	**0.005**
Extremely severe	6 (1.5)	6 (3.7)	0 (0.0)	**0.005**
Stress	7.1 ± 4.5	9.3 ± 4.5	5.5 ± 3.8	**< 0.001**
Normal	375 (94.0)	145 (88.4)	230 (97.9)	**< 0.001**
Mild	16 (4.0)	12 (7.3)	4 (1.7)	**0.008**
Moderate	8 (2.0)	7 (4.3)	1 (0.4)	**0.010**

Data presented as frequency and percentage; n (%), percentages are calculated based on available (non-missing) observations for each variable

*Abbreviations DASS* Depression, Anxiety and Stress Scale, *GERD* Gastroesophageal reflux disease,*N* total number, *PGDip* post-graduate diploma, *SD *Standard deviation

^†^Statistical differences between healthcare providers suffering from burnout and no burnout groups were tested using the Pearson's chi-squared test. Bold denotes two-sided statistical significance

^a^Other specialties included chemists and physical therapists

^b^Others included the laboratory and technical office

^c^Burnout severity is classified as moderate (50–74), high (75–99), and severe (100)

^d^For depression, normal (0–9 points), mild (10–13 points), moderate (14–20 points), severe (21–27 points). For anxiety, normal (0–7 points), mild (8–9 points), moderate (10–14 points), severe (15–19 points), and extremely severe (20+ points). For stress, normal (0–14 points), mild (15–18 points), moderate (19–25 points)

The prevalence of burnout among HCPs was 41%. Age emerged as a pivotal factor, with HCPs under 30 years old exhibiting a significantly lower prevalence of burnout compared to those aged 30 to 45 years (Prevalence ratio (PR) = 0.63, 95% CI: 0.46 to 0.86, *P* = 0.003). Furthermore, individuals aged over 45 years had a significantly diminished likelihood of burnout compared to their counterparts aged 30 to 45 years (PR = 0.50, 95% CI: 0.26 to 0.97, *P* = 0.041). Work schedule played a significant role, with night-shift workers showing a higher prevalence of burnout compared to those on morning shifts (PR = 1.38, 95% CI: 1.09 to 1.74, *P* = 0.007).

Multivariable ordinal logistic regression revealed significant differences in four out of the ten PSMA domains between HCPs suffering from burnout versus not, infection prevention and control, patient communication and education, patient safety culture, and documentation and record-keeping. Within the infection prevention and control domain, respondents experiencing burnout showed a significant increase in the odds of being suboptimal in complying with infection control guidelines (Odds ratio (OR) = 3.78, 95% CI: 1.04 to 13.69, *P* = 0.04) and a significant decrease in the odds of being familiar with standard precautions for preventing the spread of infections (OR = 0.30, 95% CI: 0.11 to 0.82, *P* = 0.02) compared to those who were not experiencing burnout. For the patient communication and education domain, burnout significantly increased the probability of being moderate in involving patients in decision-making (OR = 9.69, 95% CI: 2.53 to 37.10, *P* = 0.001). Regarding the patient safety culture domain, burnout displayed a significant association with inconsistent speaking up about patient safety concerns within teams and departments (OR = 3.62, 95% CI: 1.18 to 11.07, *P* = 0.02). For the documentation and record-keeping domain, burnout significantly increased the likelihood of sometimes maintaining accurate patient records (OR = 5.17, 95% CI: 1.22 to 21.81, *P* = 0.03). Detailed results of the crude and adjusted association between burnout and PSMA are shown in Table 2.

**Table 2:** The association between healthcare providers’ burnout and patient safety management activities within healthcare settings

Domains			Crude odds ratio		Adjusted odds ratio	
			OR (95% CI)	*P* value†	OR (95% CI)	*P* value†
1. Patient Identification and Safety						
Do you verify patient identification (e.g., name, date of birth) before administering medications or performing procedures?						
No^Ⓡ^						
Sometimes/Maybe/ Neutral			1.40 (0.54, 3.65)	0.50	1.25 (0.30, 5.29)	0.76
Yes			0.73 (0.34, 1.59)	0.44	0.59 (0.18, 2.00)	0.40
Are you familiar with the organization’s policies on patient identification and safety?						
No^Ⓡ^						
Sometimes/Maybe/ Neutral			1.35 (0.60, 3.05)	0.47	0.99 (0.32, 3.12)	0.99
Yes			0.59 (0.34, 1.04)	0.07	0.45 (0.19, 1.07)	0.07
2. Medication Safety						
Do you double-check medication dosages and administration routes before administering medications to patients?						
No^Ⓡ^						
Sometimes/Maybe/ Neutral	1.43 (0.62, 3.33)			0.41	1.59 (0.48, 5.28)	0.45
Yes	0.72 (0.37, 1.41)			0.34	1.04 (0.40, 2.73)	0.94
Are you aware of the procedures for reporting medication errors or adverse drug reactions within the organization?						
No^Ⓡ^						
Sometimes/Maybe/ Neutral			0.80 (0.35, 1.84)	0.61	0.63 (0.20, 2.02)	0.44
Yes			0.55 (0.30, 1.00)	0.05	0.44 (0.19, 1.01)	0.05
3. Infection Prevention and Control						
Do you comply with infection control guidelines, including hand hygiene and the appropriate use of personal protective equipment?						
NoⓇ						
Sometimes/Maybe/ Neutral			3.65 (1.48, 9.02)	**0.005**	3.78 (1.04, 13.69)	**0.04**
Yes			1.07 (0.52, 2.18)	0.86	1.12 (0.41, 3.11)	0.82
Are you familiar about standard precautions for preventing the spread of infections?						
No^Ⓡ^						
Sometimes/Maybe/ Neutral			0.82 (0.34, 1.99)	0.66	0.51 (0.12, 2.07)	0.34
Yes			0.51 (0.26, 0.99)	**0.04**	0.30 (0.11, 0.82)	**0.02**
4. Patient Communication and Education						
Do you involve patients in decision-making about their care and treatment options?						
No^Ⓡ^						
Sometimes/Maybe/ Neutral			4.04 (1.64, 9.98)	**0.002**	9.69 (2.53, 37.10)	**0.001**
Yes			0.85 (0.44, 1.63)	0.62	1.03 (0.42, 2.49)	0.96
Are you proactive in providing clear and comprehensive instructions to patients upon discharge?						
No^Ⓡ^						
Sometimes/Maybe/ Neutral			2.82 (1.14, 6.97)	**0.03**	2.04 (0.52, 8.03)	0.31
Yes			0.92 (0.44, 1.92)	0.83	0.45 (0.14, 1.37)	0.16
5. Adherence to Evidence-Based Clinical Guidelines						
Do you refer to evidence-based clinical guidelines or protocols in your practice?						
No^Ⓡ^						
Sometimes/Maybe/ Neutral			1.75 (0.65, 4.72)	0.27	4.41 (0.85, 22.98)	0.08
Yes			1.68 (0.75, 3.79)	0.21	3.61 (0.88, 14.85)	0.08
Do you collaborate with other healthcare team members to ensure consistent adherence to best practices?						
No^Ⓡ^						
Sometimes/Maybe/ Neutral			1.77 (0.74, 4.23)	0.19	0.84 (0.23, 3.12)	0.79
Yes			1.19 (0.58, 2.44)	0.64	0.59 (0.20, 1.77)	0.35
6. Patient Safety Culture						
Do you feel speaking up about patient safety concerns within your team or department?						
No^Ⓡ^						
Sometimes/Maybe/ Neutral			3.06 (1.35, 6.93)	**0.008**	3.62 (1.18, 11.07)	**0.02**
Yes			1.01 (0.54, 1.89)	0.98	1.13 (0.49, 2.62)	0.77
Are you encouraged to report safety incidents without fear of retribution?						
No^Ⓡ^						
Sometimes/Maybe/ Neutral			2.15 (1.00, 4.59)	0.05	2.26 (0.81, 6.31)	0.12
Yes			0.91 (0.49, 1.71)	0.77	1.10 (0.46, 2.66)	0.83
7. Performance Improvement and Quality Initiatives						
Do you participate in quality improvement initiatives or performance improvement projects within your department or unit?						
No^Ⓡ^						
Sometimes/Maybe/ Neutral			2.90 (1.32, 6.35)	**0.008**	2.24 (0.76, 6.65)	0.15
Yes			1.61 (0.83, 3.14)	0.16	1.53 (0.60, 3.91)	0.37
Are you aware of the organization’s efforts to monitor and improve patient safety and healthcare quality?						
No^Ⓡ^						
Sometimes/Maybe/ Neutral			1.79 (0.83, 3.90)	0.14	1.87 (0.64, 5.42)	0.25
Yes			1.00 (0.52, 1.93)	0.99	1.27 (0.52, 3.11)	0.60
8. Patient Rights and Privacy						
Do you respect and protect patients’ rights to privacy and confidentiality? **						
No^Ⓡ^						
Sometimes/Maybe/ Neutral			3.00 (0.90, 9.96)	0.07	17.32 (0.84, 35.38)	0.07
Yes			1.37 (0.60, 3.13)	0.46	1.13 (0.33, 3.91)	0.84
Do you ensure that patients understand their rights and have the opportunity to voice their concerns or preferences?						
No^Ⓡ^						
Sometimes/Maybe/ Neutral			1.13 (0.44, 2.87)	0.79	0.57 (0.13, 2.38)	0.43
Yes			0.81 (0.38, 1.75)	0.59	0.58 (0.18, 1.86)	0.36
9. Emergency Preparedness						
Are you familiar with the organization’s emergency response protocols and procedures?						
No^Ⓡ^						
Sometimes/Maybe/ Neutral			2.49 (0.99, 6.26)	0.05	3.32 (0.80, 13.85)	0.09
Yes			1.29 (0.63, 2.60)	0.49	1.69 (0.57, 5.04)	0.35
Have you participated in any emergency preparedness training or drills?						
No^Ⓡ^						
Sometimes/Maybe/ Neutral			2.00 (0.90, 4.46)	0.09	2.09 (0.66, 6.66)	0.21
Yes			0.88 (0.48, 1.59)	0.66	0.79 (0.35, 1.78)	0.56
10. Documentation and Record-Keeping						
Do you maintain accurate and complete patient records and documentation?						
No^Ⓡ^						
Sometimes/Maybe/ Neutral			2.64 (1.02, 6.86)	0.05	5.17 (1.22, 21.81)	**0.03**
Yes			1.48 (0.68, 3.23)	0.32	2.80 (0.83, 9.50)	0.09
Are you aware of the policies and procedures for record-keeping within the organization?						
No^Ⓡ^						
Sometimes/Maybe/ Neutral			2.16 (0.91, 5.10)	0.08	2.41 (0.64, 9.15)	0.19
Yes			1.10 (0.59, 2.06)	0.77	0.92 (0.36, 2.32)	0.85

*AbbreviationsCI* Confidence interval, *OR* Odds ratio, Ⓡ reference (1)

† Bold denotes two-sided statistical significance

Modified Poisson regression analysis aimed at assessing the adjusted effect of each potential factor contributing to practicing PSMA among HCPs while controlling for the other factors. The analysis revealed that healthcare setting and depression were risk factors associated with reduced PSMA., Table 3.

**Table 3:** Modified Poisson regression for the association between healthcare providers’ burnout and patient safety management activities and identification of potential risk factors

Predictors	PR (95% CI)	*P* value†
Age categories		
Less than 30 years old	1.03 (0.67, 1.59)	0.89
30 to 45 years old^Ⓡ^		
More than 45 years old	0.55 (0.28, 1.07)	0.08
Sex		
Male^Ⓡ^		
Female	0.96 (0.71, 1.28)	0.77
Highest level of education		
Diploma	0.24 (0.06, 1.00)	0.05
Student	1.22 (0.64, 2.34)	0.54
Bachelor^Ⓡ^		
PGDip	0.91 (0.35, 2.40)	0.85
Fellowship	0.77 (0.52, 1.14)	0.19
Master’s degree	0.90 (0.65, 1.23)	0.50
Specialty ^a^		
Physician	1.49 (0.78, 2.84)	0.23
Nurse	1.51 (0.76, 2.98)	0.24
Pharmacist^Ⓡ^		
Experience		
More than 10 years^Ⓡ^		
Between 5 to 10 years	0.90 (0.66, 1.22)	0.49
Less than 5 years	1.03 (0.68, 1.56)	0.89
Healthcare setting		
Hospital wards^Ⓡ^		
Outpatient clinics	0.78 (0.42, 1.47)	0.44
Pharmacy	0.68 (0.09, 5.06)	0.70
Long-term care facility	0.92 (0.20, 4.21)	0.92
Others ^b^	0.32 (0.13, 0.82)	**0.017**
Work schedule		
Morning	1.01 (0.69, 1.47)	0.98
Afternoon^Ⓡ^		
Night	1.21 (0.87, 1.68)	0.26
Number of night shifts per week		
More than two^Ⓡ^		
Two	0.95 (0.66, 1.36)	0.77
One	0.87 (0.66, 1.15)	0.33
None	1.29 (0.78, 2.14)	0.32
Marital status		
Married^Ⓡ^		
Divorced	0.88 (0.42, 1.86)	0.74
Single	0.79 (0.44, 1.41)	0.43
Number of children		
More than two^Ⓡ^		
One to two	1.12 (0.86, 1.46)	0.39
None	0.83 (0.47, 1.49)	0.54
Annual income		
Less than 50k SAR^Ⓡ^		
Between 50k to 100k SAR	1.17 (0.90, 1.53)	0.24
More than 100k SAR	1.05 (0.79, 1.39)	0.75
Chronic diseases	1.24 (0.91, 1.68)	0.17
DASS		
Depression	1.09 (1.05, 1.14)	**< 0.001**
Anxiety	1.02 (0.99, 1.05)	0.21
Stress	1.02 (0.98, 1.05)	0.36

*AbbreviationsCI*, Confidence interval, *PGDip* Post graduate diploma, *PR* Prevalence ratio, Ⓡ reference (1)

^†^Bold denotes two-sided statistical significance

^a^Other specialties, including chemist and physical therapists, expressed no burnout

^b^Others included the laboratory and technical office

## Discussion

In this study, we explored the association between burnout among HCPs and patient safety within a tertiary hospital in Saudi Arabia. Our findings revealed a high prevalence (41%) of burnout among healthcare providers. There was a notable association between burnout and patient safety outcomes, infection prevention and control, patient communication and education, patient safety culture, and documentation and record-keeping. These results underscore the critical impact of provider well-being on patient care outcomes.

Our findings were consistent with the results of study conducted in Saudi Arabia demonstrating that front-line healthcare workers experienced higher rates of occupational stress (43.9%) and burnout (55%) compared to second-line healthcare workers, who reported rates of 31.2% and 40.8%, respectively [24]. Additionally, a study conducted in Saudi Arabia during the COVID-19 pandemic indicated that 75% of healthcare workers experienced burnout. This emphasizes the substantial stress endured by healthcare providers during the pandemic and the negative impact of increased workloads and emotional strain on the mental health of healthcare providers and patient safety [25].

Our results are also conceptually consistent with the Saudi study by Jarrar et al., which demonstrated that hospital work conditions influence patient safety both directly and indirectly through burnout dimensions. Their findings showed that leadership support and physician engagement were associated with better perceived patient safety, whereas workload was associated with poorer safety outcomes, with burnout acting as a mediating pathway. Importantly, burnout dimensions, including emotional exhaustion and interpersonal disengagement, mediated the relationship between work conditions and patient safety outcomes. This suggests that burnout may function not only as an individual psychological response, but also as a mechanism through which unfavorable organizational conditions are translated into compromised safety performance. Therefore, examining burnout in relation to PSMA may provide a practical framework for identifying modifiable organizational and behavioral targets to improve patient safety. In the context of our study, the observed associations between burnout and suboptimal infection control practices, inconsistent patient involvement, reduced speaking up about safety concerns, and incomplete documentation may similarly reflect the downstream effect of occupational strain on safety-related behaviors. Although our cross-sectional design does not allow causal inference, these findings support the interpretation of burnout as part of a broader organizational safety pathway rather than an isolated individual-level problem [13].

The high prevalence of burnout among HCPs aged 30 to 45 years and those working night shifts aligns with earlier studies that identified age and work schedules as critical factors contributing to burnout [26]. The low prevalence among younger age, less than 30 years might be explained by that younger HCPs, particularly those early in their careers, may have less administrative and decision-making burden, reducing stress compared to their older colleagues who take on greater leadership and patient care responsibilities. Additionally, younger professionals may have greater adaptability and resilience to workplace challenges, mitigating burnout risk. Compared to mid-career HCPs (ages 30–45), they might also have fewer personal responsibilities, such as family obligations, contributing to better work-life balance. We found that the majority of healthcare professionals were male (62.4%), with a significant number having over 10 years of experience (50.1%). Previous studies have also pointed out the impact of experience and demographic factors on burnout, showing that more experienced staff demonstrate higher resilience to burnout compared to their less experienced counterpart [27].

This study aligns with the results of a comprehensive systematic review and meta-analysis, which demonstrated a strong correlation between burnout and patient safety incidents [12]. Here, we uncovered the significant association between burnout and various aspects of patient safety. We found that burnout was associated with reduced adherence to infection control protocols and knowledge of standard precautions. This was previously shown in other studies where burnout was linked to higher rates of compromised infection prevention practices [28, 29]. Furthermore, healthcare professionals facing burnout showed moderate willingness to involve patients in decision-making, echoing concerns highlighted in the literature about the negative impact of burnout on patient engagement and communication [30]. The significant association between burnout and reluctance to express patient safety concerns further highlights the notion that burnout can undermine the overall safety culture within healthcare teams. These findings may be further interpreted through the behavioral safety pathway of prosocial voice, which refers to speaking up with constructive suggestions or concerns intended to protect patients and improve organizational practice. Jarrar et al. recently demonstrated among nurses in Saudi Arabia that safety training was positively associated with prosocial voice and that prosocial voice mediated the relationship between safety training and key safety behaviors, including safety compliance and safety participation. This provides a relevant explanatory framework for our findings related to speaking up about patient safety concerns. Burnout may weaken safety culture not only by increasing emotional exhaustion, but also by reducing healthcare providers’ readiness to communicate concerns, engage in safety participation, and sustain proactive safety behaviors. Therefore, interventions to improve PSMA should not be limited to reducing workload and psychological distress, but should also include structured safety training, leadership support, and psychologically safe communication channels that encourage healthcare providers to voice patient safety concerns without fear of blame or retaliation [31].

Furthermore, the role of healthcare setting may also reflect the broader influence of the physical and organizational work environment on safety performance. Al-Bsheish et al. reported that although workplace physical environment was not directly associated with nurses’ safety compliance, it influenced safety compliance indirectly through perceived safety management commitment and safety participation. This finding is relevant to our results because burnout, healthcare setting, and depression may interact with environmental stressors to shape safety-related behaviors. Inadequate physical conditions, high workload areas, limited resources, crowding, noise, or poorly organized clinical spaces may increase psychological strain and reduce staff engagement in safety participation. Therefore, safety improvement strategies should address not only individual burnout symptoms, but also the environmental and organizational determinants that enable healthcare providers to comply with safety standards, communicate effectively, and participate in safety activities [32]. Leadership and management commitment are central components of this safety pathway. ICU nurses who perceived respect for safety were positively associated with safety compliance and safety participation. This supports the interpretation that healthcare providers are more likely to engage in safety behaviors when leadership visibly prioritizes safety, responds constructively to concerns, and demonstrates respect for staff safety needs. In the context of our findings, burnout may weaken safety culture partly by reducing healthcare providers’ engagement in speaking up, documentation, and patient communication; however, strong management commitment may buffer these effects by reinforcing trust, accountability, and participation in safety activities. Therefore, interventions targeting burnout and PSMA should include leadership-level strategies, not only individual-level resilience or training programs [33]. Adverse events and event reporting were associated with factors such as nurses’ experience, hospital size, accreditation status, and teaching status. These findings support a systems-based understanding of patient safety, in which adverse events emerge from the interaction between human factors, organizational structure, institutional resources, and safety reporting practices [34].

In our research, we conducted a comprehensive analysis to identify the factors contributing to PSMA. Our results indicated that the field of specialization, work environment in healthcare settings, and mental health status significantly influence patient safety. Interestingly, individuals with burnout who employed in non-hospital settings exhibited a lower prevalence of PSMA, emphasizing the impact of workplace environment on occupational stress. The strong association between depression scores and burnout draws special attention to the critical connection between mental health and occupational pressure. Previous studies have also highlighted the reciprocal relationship between mental health conditions and burnout, spotlighting the importance of a holistic approach to mental health support for healthcare professionals.

While our research supports much of the current literature, some studies present conflicting evidence concerning the connection between burnout and patient safety. For instance, a study published by the Journal of Patient Safety found no significant correlation between burnout levels and adverse patient safety incidents among a group of healthcare professionals, indicating that other factors may influence this relationship [35]. Another study discovered that, despite experiencing high levels of burnout, certain healthcare teams maintained strong patient safety standards, crediting this to effective teamwork and supportive leadership [36]. These diverging findings feature the complexity of the burnout-patient safety relationship and the potential impact of mitigating factors such as organizational culture and leadership. Interestingly, our study revealed that burnout was not associated with patient identification, medication safety, emergency preparedness, patients’ rights and privacy, and performance improvement. This likely reflect robust systemic safeguards (e.g., barcode scanning, electronic health records alerts) that mitigate individual cognitive fatigue effect on patient identification and medication safety. Emergency preparedness depends more on team drills and protocols than solo performance, buffering burnout effects. Patients’ rights/ privacy and performance improvement involve ethical processes less tied to real-time clinical judgment and so burnout.

Several key recommendations can be implemented to effectively address burnout and enhance patient safety within healthcare settings:

1. Implementation of Organizational Interventions: Healthcare organizations must develop and implement strategies aimed at reducing workloads, improving staffing levels, and providing essential support for healthcare professionals, especially those working night shifts.
2. Enhancement of Mental Health Support: Access to mental health resources and support for healthcare professionals is essential to alleviate the impact of burnout. Incorporating regular mental health assessments and interventions into routine healthcare practice is imperative.
3. Cultivation of a Positive Safety Culture: Fostering open communication and teamwork can significantly enrich the overall safety culture within healthcare teams. The priority should be given to training programs that encourage speaking up about patient safety concerns.
4. Professional Development and Training: Continuous professional development and training opportunities should be made available to healthcare professionals to enhance their skills and resilience. Special attention should be given to middle-age healthcare professionals (30–45 years) and those in high-risk specialties.
5. Regular Assessments: Routine assessments of burnout levels and patient safety practices are crucial components of efforts to improve healthcare quality.

our proposed strategies are designed to be broadly relevant. Their effectiveness depends on institutional policies, available resources, and workplace culture. These recommendations could be adapted to the study setting, considering existing initiatives, leadership involvement, and organizational support.

In considering the findings of this study, it’s important to acknowledge several limitations. First, due to the cross-sectional design, we were unable to draw direct causal links between burnout and patient safety outcomes. Second, the PSMA tool, while systematically grounded in the internationally recognized JCAHO NPSGs, has not undergone formal psychometric validation, including internal consistency reliability testing, and validity assessment. Third, although email invitations were sent to a randomly selected sample of eligible healthcare providers, the voluntary nature of participation may have introduced selection bias, as those who responded may systematically differ from non-respondents in their levels of burnout awareness, motivation, or patient safety engagement, potentially overestimating or underestimating the true associations observed. Fourth, the reliance on self-reported data introduces the potential for reporting bias, and raises concerns about subjectivity and social desirability bias. Moreover, several estimates in the multivariable models carry wide confidence intervals, likely attributable to smaller numbers of respondents within certain PSMA domain response categories. These wide intervals indicate reduced precision in those specific estimates and should be interpreted with appropriate caution. Additionally, the study was conducted within a single institution in a specific geographical and cultural context, which may restrict the generalizability of the findings to other settings and could be influenced by power gradient and social desirability bias.

## Conclusions

In summary, our study highlights the significant association between healthcare providers’ burnout and self-reported PSMA. The findings emphasize the necessity of comprehensive strategies to address burnout, aiming to enhance both provider well-being and patient safety standards. Prioritizing the mental health of healthcare providers is crucial for the overall effectiveness and sustainability of healthcare systems.

To thoroughly understand the relationship between burnout and patient safety, further research is required to include multiple centers and recommend multi-pronged interventions to mitigate burnout and improve patient safety. It is critical to explore the effectiveness of interventions aimed at reducing burnout and enhancing patient safety. Moreover, it is vital to gain insights into how organizational culture, leadership, and support systems can mitigate burnout and improve patient safety. Comparative studies across diverse healthcare settings and cultures could help pinpoint specific factors contributing to burnout and patient safety issues in different contexts. 

## Supplementary Information

Supplementary Material 1.

## Data Availability

The anonymized data used and analyzed in the study are available on reasonable request from the corresponding author **.**.

## Abbreviations

CBI: Copenhagen Burnout Inventory
CI: Confidence interval
DASS: Depression Anxiety Stress Scales
GERD: Gastroesophageal Reflux Disease
HCPs: healthcare providers
JCAHO: Joint Commission on Accreditation of Healthcare Organizations
N: Total Number
NPSGs: National Patient Safety Goals
OR: Odds Ratio
PGDip: Postgraduate Diploma
PR: Prevalence Ratio
PSMA: Patient Safety Management Activities
SAR: Saudi Riyal
STROBE: Strengthening the Reporting of Observational Studies in Epidemiology
