# Supplementary material for: Burnout among healthcare providers and its association with patient safety management: when exhaustion breaks safety
Source: BMC Psychol. 2026 Jun 5;14:829. doi: 10.1186/s40359-026-04890-y (PMC13242108; doi:10.1186/s40359-026-04890-y)
Supplement: Supplementary file 1 — Supplementary Material 1. [file 40359_2026_4890_MOESM1_ESM.docx]

**Burnout Among Healthcare Providers and Its Association with Patient Safety Management: When Exhaustion Breaks Safety**

Dear Participant,

We sincerely appreciate your interest in taking part in our online questionnaire on the association between healthcare provider burnout and patient safety and quality of care. This study aims to shed light on the crucial relationship between the well-being of healthcare professionals and the standard of care they deliver to patients. Your valuable insights as a healthcare provider are vital to enriching our understanding of this significant topic.

Before we proceed, we would like to inform you that participation in this study is entirely voluntary. You have the right to decline or withdraw from the questionnaire at any point without facing any consequences. Additionally, your anonymity and confidentiality will be rigorously upheld throughout the research process. All data collected will be used strictly for research purposes and will be reported in an aggregated and anonymous manner, ensuring that your identity remains protected.

By continuing with the questionnaire, you are providing your informed consent to participate in this study. We kindly request you to answer the questions honestly and to the best of your knowledge, as your responses will contribute significantly to the advancement of our understanding of healthcare provider burnout and its potential impact on patient safety and quality of care. This survey composes of 3 sections and will take about 15 minutes.

**1 - Section1: Demographic characteristics**

- Age (less than 30 years old OR 30 to 45 years old OR more than 45 years old)
- Gender (Male OR Female OR Prefer not to say)
- Highest level of education (MBBCh/ MD OR Master’s degree OR Fellowship OR PhD OR Others, please mention) Multiple choices are allowed
- Profession/ Specialty
- Experience (Less than 5 years OR 5 to 10 years OR more than 10 years)
- Healthcare setting (Hospital OR Clinic OR Long-term care facility OR Others, please mention)
- Work schedule (Morning OR Afternoon OR Night) Multiple choices are allowed
- Number of night shifts per week (1 OR 2 OR more than 2)
- Marital status (Single OR Married OR Divorced OR others)
- Number of children (Non OR 1 OR 2 OR More than 2)
- Chronic diseases (NO OR Yes, please mention)
- Annual income (Lass than 50k SAR OR 50k to 100k SAR OR More than 100k SAR)

**2 – Section2: Assessment of healthcare worker burnout**

1. **The Copenhagen Burnout Inventory (CBI)**

**CBI consists of 19 items assessing 3 different domains:**

- **Personal burnout (6 items),** assess prolonged physical and psychological exhaustion and fatigue that accumulates in a person during the day, The questions involved are more general and comprehensive from all participants irrespective of occupation.
- **Occupational/ work-related burnout (7 items),** assess prolonged physical and psychological exhaustion and fatigue that derive from work.
- **Client-related burnout (6 items),** assess prolonged physical and psychological exhaustion and fatigue that is perceived as a consequence of interpersonal relation with clients.

**You will receive a score from 0–100 on each subscale, as well as a total score that is the average of all three which is interpreted as follows:**

Threshold score: 50↑

Scores of 50–74 = moderate burnout

Scores of 75–99 = high burnout

Score of 100 = severe burnout

**- Personal burnout:** Each item is rated on a 5-point Likert scale, typically ranging from: Always (100 points), Often (75 points), Sometimes (50 points), Seldom (25 points), Never/almost never (0 points). Total score on the scale is the average of the scores on the items. If less than three questions have been answered, the respondent is classified as non-responder.

1. How often do you feel tired?

2. How often are you physically exhausted?

3. How often are you emotionally exhausted?

4. How often do you think: ”I can’t take it anymore”?

5. How often do you feel worn out?

6. How often do you feel weak and susceptible to illness?

- **Work-related burnout**: Each item is rated on a 5-point Likert scale, the three first questions: To a very high degree (100 points), To a high degree (75 points), Somewhat (50 points), To a low degree (25 points), To a very low degree (0 points). Last four questions: Always (100 points), Often (75 points), Sometimes (50 points), Seldom (25 points), Never/almost never (0 points). Reversed score for last question. Scoring as for the first scale. If less than four questions have been answered, the respondent is classified as non-responder.

1. Is your work emotionally exhausting?

2. Do you feel burnt out because of your work?

3. Does your work frustrate you?

4. Do you feel worn out at the end of the working day?

5. Are you exhausted in the morning at the thought of another day at work?

6. Do you feel that every working hour is tiring for you?

7. Do you have enough energy for family and friends during leisure time?

- **Client-related burnout**: Each item is rated on a 5-point Likert scale, The four first questions: To a very high degree (100 points), To a high degree (75 points), Somewhat (50 points), To a low degree (25 points), To a very low degree (0 points). The two last questions: Always (100 points), Often (75 points), Sometimes (50 points), Seldom (25 points), Never/almost never (0 points). If less than three questions have been answered, the respondent is classified as non-responder.

1. Do you find it hard to work with clients?

2. Do you find it frustrating to work with clients?

3. Does it drain your energy to work with clients?

4. Do you feel that you give more than you get back when you work with clients?

5. Are you tired of working with clients?

6. Do you sometimes wonder how long you will be able to continue working with clients?

1. **The DASS scale**

Item on a 4-point Likert scale, ranging from 0 (Did not apply to me at all) to 3 (Applied to me very much or most of the time)

Interpretation: The total scores for each subscale can be used to assess the severity of symptoms in the following way:

- **Depression:**
  - Normal: 0-9
  - Mild: 10-13
  - Moderate: 14-20
  - Severe: 21-27
  - Extremely severe: 28+
- **Anxiety:**
  - Normal: 0-7
  - Mild: 8-9
  - Moderate: 10-14
  - Severe: 15-19
  - Extremely severe: 20+
- **Stress:**
  - Normal: 0-14
  - Mild: 15-18
  - Moderate: 19-25
  - Severe: 26-33
  - Extremely severe: 34+

Depression (DASS-D):

1. I found it hard to wind down.
2. I was aware of dryness of my mouth.
3. I couldn't seem to experience any positive feeling at all.
4. I experienced breathing difficulty (e.g., excessively rapid breathing, breathlessness in the absence of physical exertion).
5. I found it difficult to work up the initiative to do things.
6. I tended to overreact to situations.
7. I experienced trembling (e.g., in the hands).

Anxiety (DASS-A):

1. I felt I was close to panic.
2. I was worried about situations in which I might panic and make a fool of myself.
3. I experienced trembling (e.g., in the hands).
4. I was worried about situations where I might become anxious and make a fool of myself.
5. I felt terrified.
6. I felt I was going to lose control of myself.
7. I was aware of the action of my heart in the absence of physical exertion (e.g., sense of heart rate increase, heart missing a beat).

Stress (DASS-S):

1. I found it difficult to relax.
2. I found myself getting upset rather easily.
3. I felt that I was rather touchy.
4. I was aware of dryness of my mouth.
5. I experienced trembling (e.g., in the hands).
6. I found it difficult to calm down after something upset me.
7. I felt that I was using a lot of nervous energy.

**3 – Section3: PATIENT SAFETY AND QUALITY OF CARE**

**Each item is rated on a 3-point Likert scale Yes, No, or Sometimes/May be/ Neutral**

1. **Patient Identification and Safety**

a. Do you verify patient identification (e.g., name, date of birth) before administering medications or performing procedures?

b. Are you familiar with the organization's policies on patient identification and safety?

1. **Medication Safety**

a. Do you double-check medication dosages and administration routes before administering medications to patients?

b. Are you aware of the procedures for reporting medication errors or adverse drug reactions within the organization?

1. **Infection Prevention and Control**

a. Do you comply with infection control guidelines, including hand hygiene and the appropriate use of personal protective equipment?

b. Are you familiar about standard precautions for preventing the spread of infections?

1. **Patient Communication and Education**

a. Do you involve patients in decision-making about their care and treatment options?

b. Are you proactive in providing clear and comprehensive instructions to patients upon discharge?

1. **Adherence to Evidence-Based Clinical Guidelines**

a. Do you refer to evidence-based clinical guidelines or protocols in your practice?

b. Do you collaborate with other healthcare team members to ensure consistent adherence to best practices?

1. **Patient Safety Culture**

a. Do you feel speaking up about patient safety concerns within your team or department? b. Are you encouraged to report safety incidents without fear of retribution?

1. **Performance Improvement and Quality Initiatives**

a. Do you participate in quality improvement initiatives or performance improvement projects within your department or unit?

b. Are you aware of the organization's efforts to monitor and improve patient safety and healthcare quality?

1. **Patient Rights and Privacy**

a. Do you respect and protect patients' rights to privacy and confidentiality?

b. Do you ensure that patients understand their rights and have the opportunity to voice their concerns or preferences?

1. **Emergency Preparedness**

a. Are you familiar with the organization's emergency response protocols and procedures?

b. Have you participated in any emergency preparedness training or drills?

1. **Documentation and Record-Keeping**

a. Do you maintain accurate and complete patient records and documentation?

b. Are you aware of the policies and procedures for record-keeping within the organization?
